# Serial Assessment of Therapeutic Response to a New Radiosensitization Treatment, Kochi Oxydol-Radiation Therapy for Unresectable Carcinomas, Type II (KORTUC II), in Patients with Stage I/II Breast Cancer Using Breast Contrast-Enhanced Magnetic Resonance Imaging

**DOI:** 10.3390/cancers8010001

**Published:** 2015-12-22

**Authors:** Shin Yaogawa, Yasuhiro Ogawa, Shiho Morita-Tokuhiro, Akira Tsuzuki, Ryo Akima, Kenji Itoh, Kazuo Morio, Hiroaki Yasunami, Masahide Onogawa, Shinji Kariya, Munenobu Nogami, Akihito Nishioka, Mitsuhiko Miyamura

**Affiliations:** 1 Department of Diagnostic Radiology & Radiation Oncology, Medical School, Kochi University, Nankoku, Kochi 783-8505, Japan; jm-yaogawa@kochi-u.ac.jp (S.Y.); jm-tokuhiros@kochi-u.ac.jp (S.M.-T.); jm-tsuzukia@kochi-u.ac.jp (A.T.); jm-akima.r@kochi-u.ac.jp (R.A.); kariyas@kochi-u.ac.jp (S.K.); mnogami@kochi-u.ac.jp (M.N.); nishiokaa@kochi-u.ac.jp (A.N.); 2 Hyogo Prefectural Kakogawa Medical Center, Kakogawa, Hyogo 675-8555, Japan; 3 Division of Radiology, Medical School Hospital, Kochi University, Nankoku, Kochi 783-8505, Japan; jm-itohk@kochi-u.ac.jp (K.I.); jm-moriok@kochi-u.ac.jp (K.M.); jm-yasunami@kochi-u.ac.jp (H.Y.); 4 Department of Pharmacy, Medical School Hospital, Kochi University, Nankoku, Kochi 783-8505, Japan; jm-ma_ono@kochi-u.ac.jp (M.O.); miyamus@kochi-u.ac.jp (M.M.)

**Keywords:** MRI, breast cancer, KORTUC, hydrogen peroxide, radiosensitizer, sodium hyaluronate

## Abstract

Background: We have developed a new radiosensitization treatment called Kochi Oxydol-Radiation Therapy for Unresectable Carcinomas, Type II (KORTUC II). Using KORTUC II, we performed breast-conserving treatment (BCT) without any surgical procedure for elderly patients with breast cancer in stages I/II or patients refusing surgery. Since surgery was not performed, histological confirmation of the primary tumor region following KORTUC II treatment was not possible. Therefore, to precisely evaluate the response to this new therapy, a detailed diagnostic procedure is needed. The goal of this study was to evaluate the therapeutic response to KORTUC II treatment in patients with stage I/II breast cancer using annual breast contrast-enhanced (CE) magnetic resonance imaging (MRI). Methods: Twenty-one patients with stage I/II breast cancer who were elderly and/or refused surgery were enrolled in this study. All patients underwent MRI prior to and at 3 to 6 months after KORTUC II, and then approximately biannually thereafter. Findings from MRI were compared with those from other diagnostic modalities performed during the same time period. Results: KORTUC II was well tolerated, with minimal adverse effects. All of 21 patients showed a clinically complete response (cCR) on CE MRI. The mean period taken to confirm cCR on the breast CE MRI was approximately 14 months. The mean follow-up period for the patients was 61.9 months at the end of October 2014. Conclusions: The therapeutic effect of BCT using KORTUC II without surgery could be evaluated by biannual CE MRI evaluations. Approximately 14 months were required to achieve cCR in response to this therapy.

## 1. Introduction

**Figure 1:**
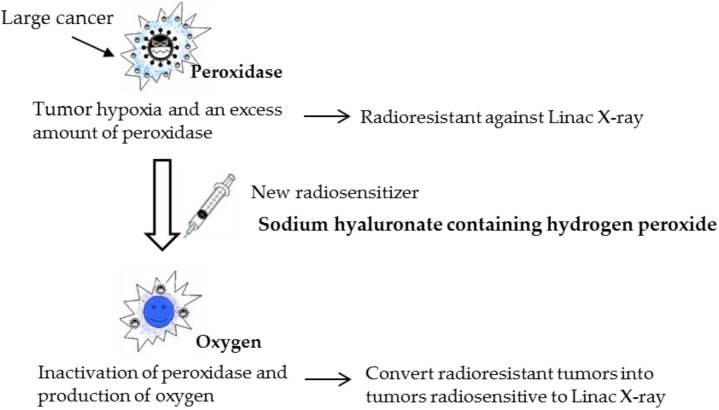
The concept of a new enzyme-targeting radiosensitization treatment (KORTUC II). Using a new radiosensitizer, various radioresistant tumors can be converted into radiosensitive tumors.

Using a current linear accelerator, our intent was to inactivate peroxidase/catalase in tumor tissue by the application of hydrogen peroxide, which is degraded to produce oxygen, thus re-oxygenating the tumor tissue [1,2,3]. In this way, we can convert tumors from radioresistant to radiosensitive. On the basis of this strategy, we had previously developed a new enzyme-targeting radiosensitization treatment named Kochi Oxydol-Radiation Therapy for Unresectable Carcinomas, Type I (KORTUC I), which markedly enhanced the radiotherapeutic effect on various types of superficially exposed and locally advanced malignant neoplasms [4]. Based on our clinical experience using KORTUC I, we also developed a new radiosensitizer containing hydrogen peroxide and sodium hyaluronate for injection into various types of tumors that are not superficially exposed [5,6,7]. The concepts underlying this new enzyme-targeting radiosensitization treatment, KORTUC II, are shown in Figure 1.

Using KORTUC II, we conducted breast-conserving therapy (BCT) without surgery for stage I/II breast cancer in elderly patients and/or those refusing surgery. Since surgery was not performed, histological confirmation of the primary tumor region following KORTUC II treatment was not possible. Instead of histological confirmation, breast contrast-enhanced (CE) magnetic resonance imaging (MRI), which is considered highly useful for precise evaluation of therapeutic responses to neo-adjuvant chemotherapy (NAC) or induction chemotherapy for patients with breast cancer, was used [8,9]. Therefore, the purpose of this study was to use biannual CE MRI to evaluate the therapeutic response to KORTUC II treatment and the time required to achieve clinically complete response (cCR) in patients with stage I/II breast cancer who were treated with KORTUC II.

## 2. Results

**Figure 2:**
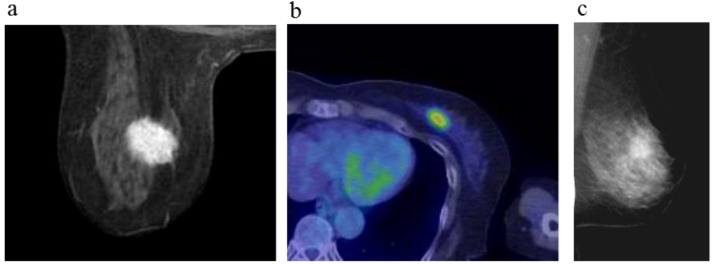

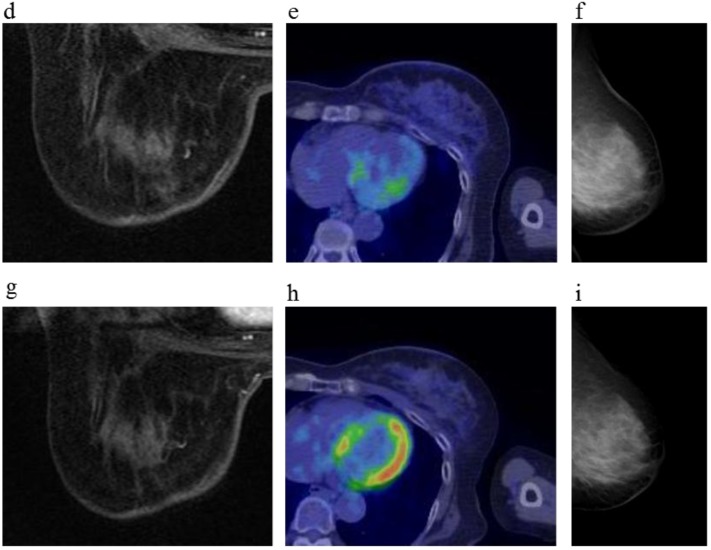
A 64-year-old female (case 3 in Table 1, Table 2 and Table 3) with left breast cancer (cT2N0M0). CE breast MRI, PET-CT and mammography revealed a breast tumor before KORTUC II therapy, the region of tumor was >20 mm (**a**–**c**). After the completion of KORTUC II, the tumor disappeared on the serial examination ((**d**–**f**): initial examination after KORTUC II treatment, (**g**–**i**): final examination), and no recurrence was identified. The findings of CE breast MRI seems to be most reliable among these three diagnostic modalities in terms of recognition of tumor disappearance.

**Figure 3:**
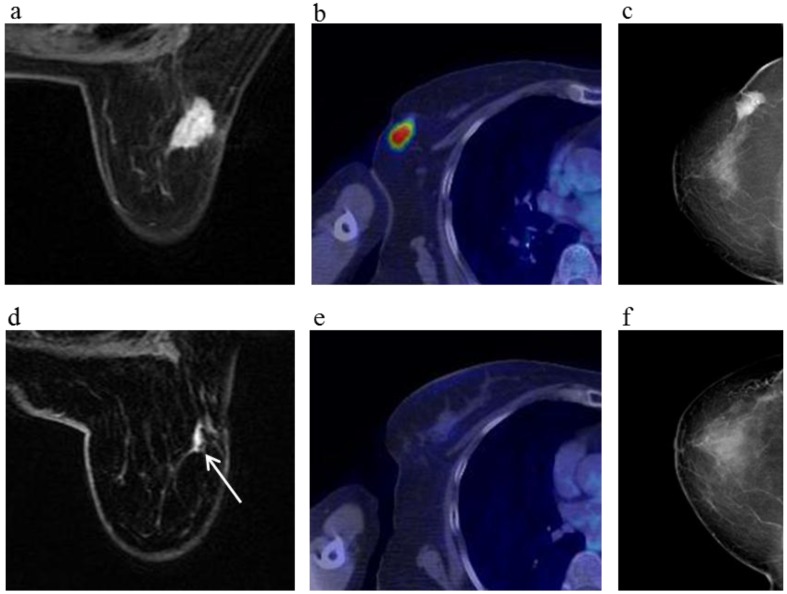

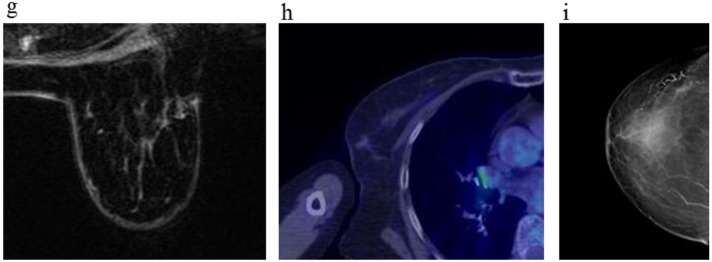
A 81-year-old female (case 7 in Table 1, Table 2 and Table 3) with right breast cancer (cT2N1M0). CE breast MRI, PET-CT and mammography revealed a breast tumor before KORTUC II therapy, the region of tumor was 25 mm in large diameter (**a**–**c**); At the initial examination after the completion of KORTUC II treatment, the tumor was undetectable on PET-CT and mammography (**e**,**f**); On the other hand, the residual tumor was still recognized on breast CE MRI ((**d**): arrows). However, complete response was eventually achieved and no recurrence was identified on final examinations of all of these three diagnostic modalities (**g**–**i**).

**Figure 4:**
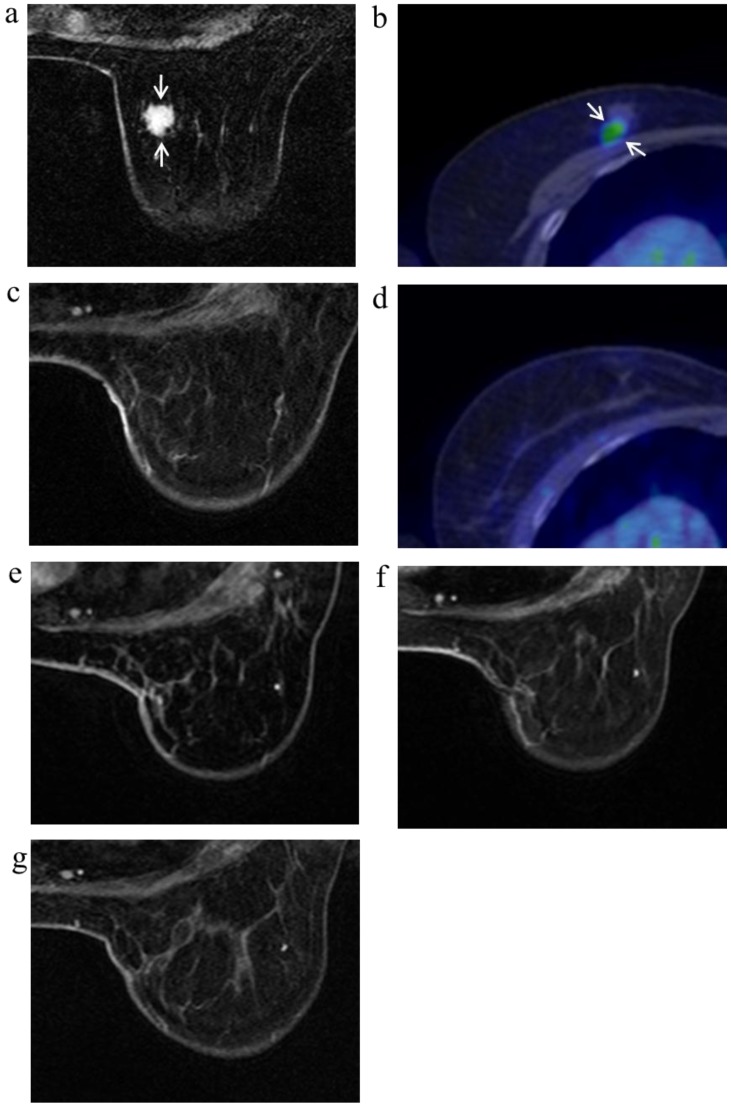
A 63-year-old female (case 8 in Table 1, Table 2 and Table 3) with right breast cancer (cT1cN0M0). CE breast MRI (**a**) and FDG-PET-CT (**b**) revealed a breast tumor before KORTUC II therapy (arrows), the region of tumor was 13 mm; After KORTUC II, no recurrence was identified on CE breast MRI and FDG-PET-CT ((**c**): 10 months; (**d**): 18 months; (**e**): 22 months; (**f**): 34 months; (**g**): 50 months).

**Figure 5:**
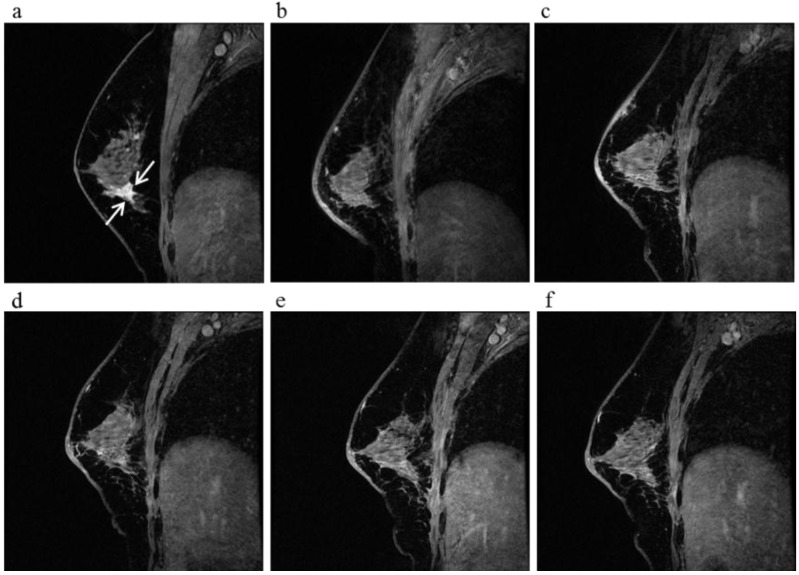
A 60-year-old female (case 4 in Table 1, Table 2 and Table 3) with right breast cancer (cT1cN0M0). CE breast MRI (**a**) revealed a breast tumor before KORTUC II therapy, the region of tumor was 18 mm (arrows); After KORTUC II, no recurrence was identified on CE breast MRI ((**b**): 5 month; (**c**): 17 month; (**d**): 29 month; (**e**): 41 months; (**f**): 53 months).

Patient data are summarized in Table 1, Table 2 and Table 3. Histological subtypes of the breast cancer of the each individual patients are shown in Table 2. KORTUC II treatment was well tolerated, with minimal adverse effects. None of the patients experienced severe complications. The mean follow-up period for patients at the end of October 2014 was 61.9 months, at which time 20 (95.2%) of 21 patients had maintained cCR on CE MRI study, and they were alive without any distant metastases. Furthermore, cCR was also confirmed through other diagnostic modalities performed during the follow-up period. Actually, the therapeutic effect was evaluated by PET-CT [10], breast MRI, ultrasonographic examination [11], and mammography [12] at approximately 4 months and approximately 10 months after KORTUC II, and these examinations were repeated biannually for at least 5 years after KORTUC II treatment, thereafter. Only one patient (case 5 in Table 1, Table 2 and Table 3) had local recurrence, which was discovered at 39 months; this patient died after 83 months of KORTUC II. Representative cases are shown in Figure 2, Figure 3, Figure 4 and Figure 5.

**Table 1:** Profiles of all patients.

Case	Age (Years)	Disease Site	TMN Stage	Endocrine Therapy
1	81	Right	cT2N0M0, stage IIA	+
2	41	Right	cTisN0M0, stage 0	+
3	64	Left	cT2N0M0, stage IIA	+
4	60	Right	cT1cN0M0, stage I	−
5	79	Left	cT2N0M0, stage IIA	+
		Right	cT1cN0M0, stage I	+
6	77	Right	cT1cN0M0, stage I	+
7	81	Right	cT2N1M0, stage IIB	+
8	63	Right	cT1cN0M0, stage I	+
9	73	Left	cT1cN0M0, stage I	+
10	61	Right	cT1cN0M0, stage I	+
11	77	Right	cT2N0M0, stage IIA	+
12	59	Left	cT2N0M0, stage IIA	+
13	50	Right	cT1cN0M0, stage I	+
14	43	Right	cT1cN0M0, stage I	+
15	77	Left	cT1cN0M0, stage I	+
16	83	Left	cT1cN0M0, stage I	+
17	63	Right	cT1cN0M0, stage I	+
18	59	Right	cT1cN0M0, stage I	+
19	77	Right	cT1cN0M0, stage I	+
20	37	Right	cT1bN0M0, stage I	+
21	49	Left	cT2N0M0, stage IIA	+

**Table 2:** Histological characteristics of breast cancer.

Case	ER/PgR	HER-2	Ki-67 Index	Histological Grade
1	+/+	2+	1%	2
2	+/+	−	1%	2
3	+/+	2+	N.A.	2
4	−/−	2+ (FISH+)	N.A.	2
5	+/+	1+	20%	1
	+/+	1+	15%	1
6	+/+	3+	10%	1
7	+/+	2+	N.A.	1
8	+/+	1+	20%	2
9	+/+	2+	N.A.	1
10	+/+	−	N.A.	1
11	+/+	2+	10%	2
12	+/+	3+	20%	2
13	+/+	2+ (FISH-)	N.A.	2
14	+/+	1+	N.A.	2
15	+/+	−	N.A.	1
16	+/+	−	30%	2
17	+/+	1+	10%	1
18	+/+	1+	10%	2
19	+/+	1+	20%	2
20	+/+	−	20%	1
21	+/+	1+	27%	1

**Table 3:** Summarized data for all patients.

Case	Assessed by MRI ^a^	Assessed by	Assessed by	Prognosis	Follow up Period
		Mammography ^a^	PET-CT ^a^		(Months)
1	cCR → cCR	cCR → cCR	N.A. → N.A.	Alive	60
2	cCR → cCR	N.A. → N.A.	cCR → cCR	Alive	70
3	cCR → cCR	cCR → cCR	cCR → cCR	Alive	73
4	cCR → cCR	cCR → cCR	cCR → cCR	Alive	66
5(Left)	cPR → cPD	cCR → cPD	cCR → cPD	Dead	83
5(Right)	cPR → cCR	cCR → cCR	cCR → cCR		
6	cCR → cCR	cCR → cCR	cCR → N.A.	Alive	78
7	cPR → cCR	cCR → cCR	cCR → cCR	Alive	68
8	cCR → cCR	cCR → cCR	cCR → cCR	Alive	73
9	cCR → cCR	cCR → cCR	cCR → cCR	Alive	79
10	cCR → cCR	cCR → cCR	cCR → cCR	Alive	68
11	cPR → cCR	cPR → cCR	cPR → cCR	Alive	78
12	cPR → cCR	cPR → cCR	cCR → cCR	Alive	92
13	cCR → cCR	cCR → cCR	cCR → cCR	Alive	60
14	cCR → cCR	cCR → cCR	cCR → cCR	Alive	56
15	cCR → cCR	cCR → cCR	cCR → cCR	Alive	56
16	cPR → cCR	cPR → cCR	cCR → cCR	Alive	39
17	cCR → cCR	cCR → N.A.	cCR → cCR	Alive	35
18	cPR → cCR	cCR → cCR	cCR → cCR	Alive	44
19	cPR → cCR	cCR → N.A.	cCR → N.A.	Dead ^b^	36
20	cCR → cCR	cCR → N.A.	cCR → cCR	Alive	40
21	cCR → cCR	cCR → N.A.	cCR → cCR	Alive	41

*cCR* clinically complete response, c*PR* clinically partial response, c*PD* clinically progressive disease. ^a^ Tumor response of the primary tumor assessed by breast CE MRI, mammography, or PET-CT (initial examination after KORTUC II treatment → final examination); ^b^ The cause of death is cardiac failure.

**Figure 6:**
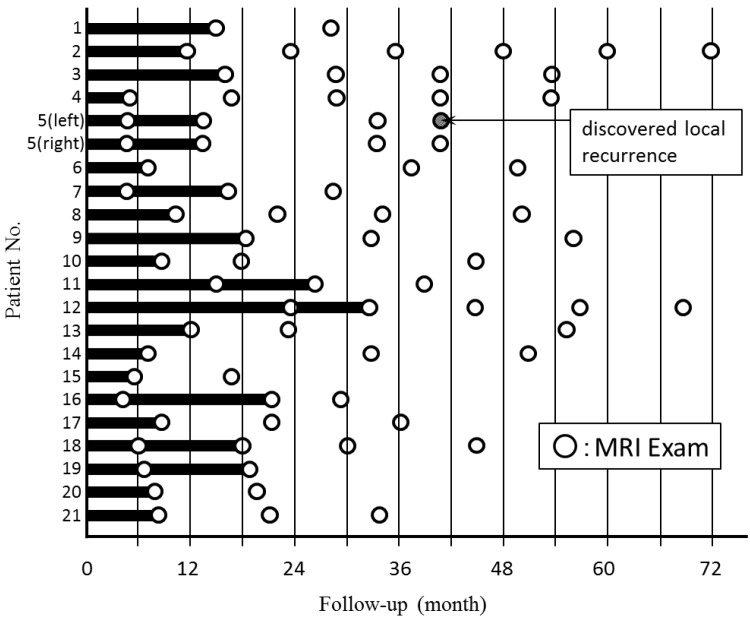
The timing of undergoing breast CE MRI after KORTUC II treatment and the period taken to accept a marked therapeutic effect of cCR on MRI. The mean period of 20 patients except case 5 was approximately 14 months.

**Figure 7:**
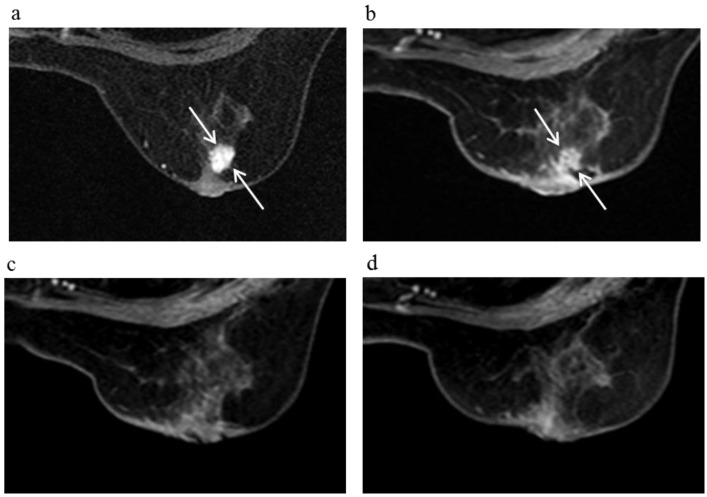
A 59-year-old female (case 18 in Table 1, Table 2 and Table 3) with right breast cancer (cT1cN0M0). CE breast MRI (**a**) revealed a breast tumor before KORTUC II therapy, the region of tumor was 11 mm (arrows). 6 months after the completion of KORTUC II, the tumor does not still disappear on breast CE MRI ((**b**): arrows); However, after 18 months (**c**) and 30 months (**d**), a complete response was achieved and no recurrence was identified on CE breast MRI.

Figure 6 shows the timing of breast CE MRI after KORTUC II treatment and shows the period taken to confirm a marked therapeutic effect of cCR on MRI. The disappearance of tumor was not always shown on the first MRI after KORTUC II, and there were seven patients whose primary tumors disappeared on the second MRI study following KORTUC II treatment. The imaging findings of a representative case are shown in Figure 7. The mean period taken to confirm cCR on the breast CE MRI for 20 patients, except case 5, was approximately 14 months.

As for comparison of the findings of breast CE MRI with those of mammography and PET-CT shown in Table 3, any significant difference in diagnostic acuity was not identified concerning the studies performed as final examinations. Though, in seven of the 21 patients examined in the study, residual tumors were recognized on the findings of breast CE MRI at initial examinations performed at several months following the KORTUC II treatment. These residual tumors eventually disappeared on the second MRI study.

## 3. Discussion

The new enzyme-targeting radiosensitization treatment, KORTUC II, enables treatment of patients with stage I/II breast cancer without any surgery, which is essentially non-surgical BCT (KORTUC-BCT). However, in the absence of surgery, histological confirmation of the primary tumor region following KORTUC II treatment cannot be obtained. Therefore, precise evaluation of the therapeutic response to this new therapy requires a detailed diagnostic procedure, such as CE MRI, PET-CT, power-Doppler ultrasonography, and/or dedicated mammography. CE MRI using a three-dimensional fast T1-weighted gradient-echo sequence with a fat suppression technique provides a high spatial resolution that is important to resolve morphologic and architectural details of tumors and to determine the extent of intraductal components of breast cancer [13]. At the same time, this fast imaging enables MR image acquisition during an optimal time window, yielding favorable tumor/breast tissue contrast, and it provides the high temporal resolution that is required for reliable evaluation of kinetic profiles of tumors [14]. Concerning diagnostic ability of MRI study for breast cancer, high sensitivity of 92.6% and high specificity of 98.4% which are obviously better than those of mammography and ultrasonography were reported [15]. Therefore, breast CE MRI is a very useful modality for the detection and characterization of breast cancer and for the evaluation of local tumor extent. In fact, breast CE MRI might be the most suitable method for confirming clinical response to the KORTUC II radiosensitization treatment in patients with breast cancer. Recently, recognition of the utility of breast MRI increased in Japan due to new guidelines concerning the treatment of breast cancer in Japan [16]. Now, breast MRI is in widespread clinical use in Japan for various purposes, including differentiating between benign or malignant lesions, yielding data that can be used to determine whether BCT is indicated, and for the evaluation of the response to neoadjuvant chemotherapy [17]. In the study, in terms of comparison of the findings of breast CE MRI with those of mammography and PET-CT shown in Table 3, any significant difference in diagnostic acuity was not identified concerning the studies performed as final examinations. Though, in seven of the 21 patients examined in the study, residual tumors were recognized on the findings of breast CE MRI at initial examinations performed at several months following the KORTUC II treatment. These residual tumors eventually disappeared on final examinations of all of these three diagnostic modalities, excluding that of case 5. There results possibly reflect the characteristics of CE MRI in terms of a high spatial resolution.

Since long-term control rates in response to KORTUC II remain unknown, close follow-up is needed for patients with dedicated diagnostic modalities. In the present study, no evidence of disease (NED) was achieved in 20 (95.2%) of 21 patients during the follow-up period (mean, 61.9 months). These results are almost equivalent to those seen in response to more common treatment methods with surgery for stage I/II breast cancer. Although BCT generally includes surgery, these results suggest that non-surgical BCT consisting of radiation therapy alone can be performed by using this new KORTUC II radiosensitization treatment. On the other hand, the mean period taken to confirm cCR on the breast CE MRI was approximately 14 months in the present study. Furthermore, the disappearance of tumor was not always shown on the first MRI study, which was performed approximately 3 to 6 months after the treatment, and there were several patients whose absence of tumor was confirmed on the second MRI study performed at approximately 1.5 years after the treatment. In contrast to breast cancer patients treated with surgery whose tumors were totally resected, almost all breast cancer patients treated with KORTUC II without surgery were apprehensive regarding their response to therapy. Non-surgical treatments typically require a relatively long period (*i.e.*, several months or more) to confirm cCR on radiological imaging modalities, because dead cancer cells require time for necrosis and reabsorption.

We believe that non-surgical breast cancer treatment, such as KORTUC II, will soon be used routinely worldwide for preservation of quality of life (QOL), improvement in breast cosmesis, and reduction of medical costs. Therefore, it is essential to establish a precise follow-up method using a dedicated imaging modality, such as CE MRI, and to determine the duration required until disappearance of the tumor following non-surgical BCT using KORTUC II radiosensitization treatment.

## 4. Patients and Methods

The study was performed at Kochi Medical School Hospital from October 2006 to April 2011. Twenty-one patients (22 lesions) with stage I/II breast cancer who were elderly and/or refused surgery were enrolled in the KORTUC II trial upon providing fully informed, written consent. Patient data are summarized in Table 1, Table 2 and Table 3. Patient age ranged from 37 to 83 years (mean 64.5 years). According to the TMN classification, the clinical stages of the patients were 0 (*n* = 1), I (*n* = 13), IIA (*n* = 6), and IIB (*n* = 1). A risk category was assigned to each patient according to the updated St.Gallen consensus based on clinical tumor size and the pathological results of a core needle biopsy taken before therapy. From the needle biopsy specimen obtained at pre-treatment, hormonal status (estrogen and progesterone receptors), HER-2 antigen, the Ki-67 index, and CD44 receptor status were examined by immunohistochemistry. CD44-positive tumor cells have been reported to be breast cancer stem cells, and these cells might migrate into lymphatic vessels if sodium hyaluronate alone were injected into tumor tissue. Since hydrogen peroxide is utilized in KORTUC II treatment, the partial oxygen pressure in hypoxic breast cancer stem cells can be increased, and the radio-resistance of breast cancer stem cells under hypoxic circumstances is converted into a radio-sensitive state.

Chemotherapy was not administered before or after KORTUC II treatment. Concerning endocrine therapy, all patients with hormone receptor-positive breast tumors received endocrine therapy immediately after the completion of KORTUC II treatment. Tamoxifen (20 mg/day per os) or an aromatase inhibitor (anastrozole 1 mg/day or exemestane 25 mg/day per os) was used for premenopausal and postmenopausal patients, respectively. Endocrine therapy was scheduled to continue for 5 years in all eligible patients.

For these patients, radiation therapy with 4-MV X-rays was delivered with an EXL-20TP linear accelerator equipped with a multi-leaf collimator (Mitsubishi Electric Co., Ltd., Tokyo, Japan). Hypofractionated radiation therapy was administered using a tangential field approach with a field-in-field method including an ipsilateral axillary region; the energy level was 4 MV, and the total dose was 44 Gy, administered as 2.75 Gy/fraction. Radiation therapy was performed five times per week for each patient. Boost irradiation was delivered using an electron beam of appropriate energy for each individual patient and was administered concurrently with a dose of 9 Gy in three fractions in the last week of radiation therapy with 4-MV X-ray [18,19].

The new radiosensitizer was injected into the breast tumor tissue twice a week under ultrasonographic guidance, just prior to each administration of radiation therapy from the 6th fraction onwards. The agent is composed of 0.5% hydrogen peroxide and 0.83% sodium hyaluronate, which is safe for injection and effectively preserves oxygen concentration in the tumor tissue for more than 24 h following intratumoral injection [7]. Concerning the radiosensitizer, a syringe (2.5 mL) of a hyaluronic acid preparation having a 1% *w*/*v* concentration of sodium hyaluronate (ARTZ Dispo, Seikagaku Corporation, Tokyo, Japan) was used. This contained 25 mg of sodium hyaluronate, 2.5 mg of l-methionine, sodium chloride, potassium phosphate, crystalline sodium dihydrogen phosphate, and an isotonizing agent. The preparation is a colorless, transparent, viscous, aqueous solution having a pH of 6.8 to 7.8, specific osmotic pressure of 1.0 to 1.2 (relative to physiological saline), and a weight-average molecular weight of 600,000 to 1.2 million. To this, 0.5 mL of a 3% *w*/*v* solution of hydrogen peroxide (Oxydol, Ken-ei Pharmaceutical Co. Ltd., Osaka, Japan) was added immediately before use and mixed well to prepare the radiosensitizer. Hydrogen peroxide, as a small vial containing 0.5 mL of 3% hydrogen peroxide, was aseptically subdivided into the vial using a Milipore-filter and kindly provided to us by the Department of Pharmacy, Kochi Medical School Hospital. The sensitizer has a sodium hyaluronate concentration of 0.83% and a hydrogen peroxide concentration of approximately 0.5%. This preparation was used in the study.

All patients underwent breast CE MRI prior to and at approximately 3 to 6 months after KORTUC II treatment and then approximately biannually thereafter. Findings from MRI were compared with those from [^18^F]-fluorodeoxyglucose positron emission CT (FDG-PET-CT) performed during the same time period. All MRI scans were performed on a 3-T scanner (Signa HDx: GE Healthcare, Milwaukee, WI, USA). The patients were examined in the prone position using a bilateral phased-array breast coil. They underwent dynamic study after pre-gadolinium sequences. Dynamic study was performed by Volume Imaged Breast AssessmeNT (VIBRANT) using a three-dimensional (3D) fast spoiled gradient-echo sequence in the axial plane and the following parameters: repetition time (TR) = 7.2 msec, echo time (TE) = 4.3 msec, flip angle (FA) = 10°, field of view (FOV) = 36 × 36 cm^2^, matrix size = 512 × 256 pixels, slice thickness = 3 mm, gap = 0 mm, and number of excitations (NEX) = 0.7. In the dynamic study, serial examinations were performed before and 8 times (every 30 s) after bolus injection of 0.1 mmol gadolinium diethylenetriamine pentaacetic acid (Gd-DTPA)/kg by automatic injector (Medrad, Pittsburgh, PA, USA) at a rate of 3 mL/sec, followed by a 30-ml saline flush. After dynamic imaging, CE T1-weighted fat-suppressed sagittal images using a 3D fast spoiled gradient-echo sequence were obtained. Scan parameters were TR = 16.9 msec, TE = 4.3 msec, FA = 20°, FOV = 20 × 20 cm^2^, matrix size = 512 × 256 pixels, slice thickness = 1.5 mm, gap = 0 mm, and NEX = 1.2. Whole-body FDG-PET-CT scans were obtained on a Discovery ST Elite PET-CT system (GE Healthcare) consisting of a full ring dedicated PET and a 16-slice spiral CT. All patients were instructed to fast for 6 h before the examination. The examination was initiated approximately 60 min after injection of 3.5 MBq/kg FDG. CT was acquired before PET with a 5-mm slice thickness and a 3.7-mm increment. After CT, a 3D mode PET was performed. The PET emission time per bed position was adapted to patient body weight: <65 kg, 2 min per bed position; 65–85 kg, 2.5 min; and >85 kg, 3 min.

The interpreter of MRI (Munenobu Nogami) was provided with information regarding tumor location, but was blinded to undergoing therapy.

## 5. Conclusions

BCT without surgery can be safely performed using our new KORTUC II radiosensitization treatment for topical injection into the tumor tissue. KORTUC-BCT has great potential to become a viable noninvasive replacement for surgical BCT. In addition, the present study showed that breast CE MRI was useful for initial and follow-up evaluations, before and after non-surgical breast cancer treatment using KORTUC II. Dedicated diagnostic imaging is essential for the follow-up of patients who are treated with non-surgical therapy, such as KORTUC II. Among various diagnostic modalities, MRI has powerful potential as well as histological diagnosis; therefore, randomized clinical trials of KORTUC-BCT utilizing CE MRI for initial and follow-up evaluations are expected in the near future, 
